# Prevalence of STEC virulence markers and *Salmonella* as a function of abiotic factors in agricultural water in the southeastern United States

**DOI:** 10.3389/fmicb.2024.1320168

**Published:** 2024-05-20

**Authors:** Zoila R. Chevez, Laurel L. Dunn, Andre L. B. R. da Silva, Camila Rodrigues

**Affiliations:** ^1^Department of Horticulture, Auburn University, Auburn, AL, United States; ^2^Department of Food Science and Technology, University of Georgia, Athens, GA, United States

**Keywords:** agricultural water, produce safety, irrigation, *Escherichia coli*, *Salmonella enterica*

## Abstract

Fresh produce can be contaminated by enteric pathogens throughout crop production, including through contact with contaminated agricultural water. The most common outbreaks and recalls in fresh produce are due to contamination by *Salmonella enterica* and Shiga toxin-producing *E. coli* (STEC). Thus, the objectives of this study were to investigate the prevalence of markers for STEC (*wzy, hly*, *fliC*, *eaeA*, *rfbE*, *stx*-I, *stx*-II) and *Salmonella* (*invA*) in surface water sources (*n* = 8) from produce farms in Southwest Georgia and to determine correlations among the prevalence of virulence markers for STEC, water nutrient profile, and environmental factors. Water samples (500 mL) from eight irrigation ponds were collected from February to December 2021 (*n* = 88). Polymerase chain reaction (PCR) was used to screen for *Salmonella* and STEC genes, and *Salmonella* samples were confirmed by culture-based methods. Positive samples for *Salmonella* were further serotyped. Particularly, *Salmonella* was detected in 6/88 (6.81%) water samples from all ponds, and the following 4 serotypes were detected: Saintpaul 3/6 (50%), Montevideo 1/6 (16.66%), Mississippi 1/6 (16.66%), and Bareilly 1/6 (16.66%). *Salmonella* isolates were only found in the summer months (May-Aug.). The most prevalent STEC genes were *hly* 77/88 (87.50%) and *stx*-I 75/88 (85.22%), followed by *fliC* 54/88 (61.63%), *stx*-II 41/88 (46.59%), *rfbE* 31/88 (35.22%), and *eaeA* 28/88 (31.81%). The *wzy* gene was not detected in any of the samples. Based on a logistic regression analysis, the odds of codetection for STEC virulence markers (*stx*-I, *stx*-II, and *eaeA*) were negatively correlated with calcium and relative humidity (*p* < 0.05). A conditional forest analysis was performed to assess predictive performance (AUC = 0.921), and the top predictors included humidity, nitrate, calcium, and solar radiation. Overall, information from this research adds to a growing body of knowledge regarding the risk that surface water sources pose to produce grown in subtropical environmental conditions and emphasizes the importance of understanding the use of abiotic factors as a holistic approach to understanding the microbial quality of water.

## Introduction

The consumption of fruits and vegetables has significantly increased over the past few decades as people have become more health-conscious and aware of the benefits of a balanced diet (Machado-moreira et al., 2019; Balali et al., 2020). Fresh produce is often consumed raw and without a cooking step, which increases the risk of foodborne infections (Falardeau et al., 2017; Quintanilla Portillo et al., 2022). The increase in fruit and vegetable production also increases the number of recalls and foodborne outbreaks associated with fresh produce that are commonly reported due to microbial contamination (Wadamori et al., 2017). During crop production, several factors can contribute to microbial contamination, including animals as natural carriers of enteric pathogens or humans if good agricultural practices are not properly followed. Those pathogens can end up in the soil (i.e., manure) and agricultural water (leakage of septic tanks) (Strawn et al., 2013b). Particularly, water is essential for plant development, and the monitoring of microbial quality, as well as good agricultural practices, is critical to minimize risks associated with microbial contamination. This is relatively potentiated with the establishment of food safety practices to protect the safety of fresh produce (Truitt et al., 2018; Devarajan et al., 2023). Previous studies have investigated the prevalence of foodborne pathogens in irrigation water sources for vegetable crops (McEgan et al., 2013; Strawn et al., 2013b; Falardeau et al., 2017; Topalcengiz et al., 2017; Truitt et al., 2018; Weller et al., 2020a, 2020b; Belias et al., 2021; Murphy et al., 2022). Although there is a substantial body of research available, the existing studies exhibit a notable degree of inconsistency, often contradicting each other, especially when evaluating abiotic factors and pathogen prevalence. Our research seeks to better understand the use of abiotic factors as predictors of pathogens by using machine learning models and, more specifically, in subtropical environmental conditions characterized by frequent rainfall events during hot summers and dry periods during the winter, which can significantly influence crop management practices and pathogens’ survival.

Surface water is the most susceptible water source used in irrigation systems to contamination with biological hazards, including enteric pathogens like *Salmonella enterica* (*S. enterica*) and *Escherichia coli* (*E. coli*) (Rodrigues et al., 2020; Gurtler and Gibson, 2022). Once contaminated water is used in the production of fresh produce, there is a significant food safety risk and a public health concern. Finding the sources of bacterial contamination in produce whenever an outbreak occurs can be complex due to several factors, including the short shelf life of the produce, the need for patients to recall their food consumption history, and tracing back the origin of the bacteria strain recovered from the patient back to its source (e.g., water), therefore, addressing produce contamination remains challenging. Nonetheless, agricultural water has been identified as a source of contamination in numerous outbreaks and from a variety of produce, including tomatoes, cucumbers, and leafy greens (Ackers et al., 1998; Greene et al., 2008; Laughlin et al., 2019; CDC, 2019a,b). Water has been associated with two of the most recent significant outbreaks in the United States linked to fresh produce. One of these outbreaks was caused by *Salmonella* Newport infections linked to onions (CDC, 2020) that sickened over 1,000 people, while the other was caused by *E. coli* O157:H7 infections associated with romaine lettuce that sickened over 200 people (CDC, 2019b).

The southeastern United States is an important vegetable production region, especially the state of Georgia, contributing to 2.8 billion dollars to the state’s economy. Some of the most common vegetables grown in the study region include cucumbers, bell peppers, broccoli, watermelons, onions, and other fresh produce (Kane, 2023). To provide water for the extensive vegetable cultivated area, Georgia relies on a daily irrigation demand of over two billion liters, of which surface water is one of the primary sources (Painter, 2019; Kane, 2023). Previous studies on the microbial quality of water have been conducted across Georgia (Jenkins et al., 2012; Antaki et al., 2016; Harris et al., 2018) and other southern states, including Florida (Strawn et al., 2014; Murphy et al., 2022) and Virginia (Truitt et al., 2018; Gu et al., 2019). None of these studies implemented source tracking methodologies and only credited the presence of *Salmonella* in water due to factors like geography, land use, ecology, and seasonality (Rajabi et al., 2011; Li et al., 2014; Luo et al., 2015; Maurer et al., 2015).

Further studies have assessed the presence and concentration of *Salmonella,* Shiga toxin-producing *Escherichia coli* (STEC), and generic *E. coli* in irrigation water in Southern Georgia and Virginia and analyzed the relationship with different environmental factors, including total rainfall, pH, water temperature, dissolved oxygen concentration, oxidation–reduction potential, turbidity, and humidity (Gu et al., 2013, 2019; Antaki et al., 2016). In water sources from Georgia, Antaki et al. (2016) did not find correlations between generic *E. coli and Salmonella.* On the contrary, Gu et al. (2013) indicated positive correlations between *E. coli* O157 and fecal coliforms, temperature, and rainfall. Similar results were reported in Virginia, where *Salmonella* increased after the rising of air temperature and the presence of rainfall events (Gu et al., 2019).

All these aforementioned studies have identified the prevalence of pathogens and correlations with environmental factors; however, only one study has evaluated the codetection of virulence factors of STEC (Gu et al., 2013), and none of the previous studies have evaluated the genetic markers (*fliC, wzy, rfbE,* and *hly*) with a wide range of nutrients and environmental factors in ponds used for irrigation. Understanding the behavior and prevalence of STEC and *Salmonella* with abiotic factors at the farm level can be beneficial for creating predictive models. Using physicochemical data in conjunction with microbial testing can provide insights into the potential presence of enteric pathogens in water and support science-based decision-making processes. In addition, corrective actions (i.e., use of microbial die-off, water treatment, or suspension of water use) can be taken in advance to prevent and reduce pathogen contribution to fresh produce. Considering that STEC and *Salmonella* remain common etiological agents for produce-related outbreaks (Belias et al., 2021). Implementing mitigation strategies for microbial prediction is critical to protecting public health. Recognizing that several water sources in the southeastern United States have been reported to be contaminated with foodborne pathogens, it is relevant to identify factors associated with the prevalence of foodborne pathogens in these water sources. Thus, the objectives of this study were (1) to investigate the prevalence of foodborne pathogens (STEC and *Salmonella*) in surface water sources from a large produce-growing area in the Southeastern United States and (2) to determine the correlation among the prevalence of foodborne pathogens, fecal indicator bacteria, and abiotic factors.

## Materials and methods

### Irrigation ponds and water sampling

Eight irrigation ponds from large produce farms located in Southwest Georgia, United States, were selected for water sampling. All ponds were located within a 32-km radius of each other. The agricultural and physical characteristics of each pond area are described in Table 1. Surface water samples (500 mL) were collected monthly with a sterile amber glass bottle and transported to the laboratory in a cooler with ice. Water samples were collected from February to December 2021 (*n* = 88). A volume of 150 mL from each sample was filtered via the membrane filtration method through a 47 mm diameter, 0.45 μm pore-size sterile filter (Pall Corporation, Ann Arbor, Michigan, United States) as described by Topalcengiz et al. (2017). Filters were placed in Whirl-Pak bags and stored at −80°C (no cryo-protectant was used), until further processing for identification of *Salmonella* and STEC markers. Enumeration of generic *E. coli* was conducted by using the IDEXX Colilert with Quanti-Tray^®^ 2000 (IDEXX Laboratories, Westbrook, ME, United States) method, as described by Haley et al. (2022).

**Table 1:** Characteristics of the eight irrigation ponds surveyed for STEC markers and *Salmonella* in Southwest Georgia.

Pond ID	Pond size (m^2^)	Irrigation type	Crops	Distance to asphalt road (m)	Distance to houses/packing shed (m)	Immediate area land use (s)
A1	13,858.98	Drip	S, E, P, K	250	300	Agriculture
A2	19,476.42	Drip	Co	400	300	Agriculture
A3	11,107.53	Drip	Co	125	50	Agriculture
A4	11,866.82	Drip	Co	50	200	Agriculture
B1	39,823.98	Drip, pivot	BP, S, T, W	400	500	Agriculture, Forest
B2	5,679.47	Drip	K, W, M	187	350	Agriculture, residential
B3	6,172.82	Drip, pivot	Br, S, K	260	300	Agriculture, residential
B4	11,989.26	Drip	P, E, Br	50	15	Agriculture, residential

K, kale; W, watermelon; M, melons; BP, bell pepper; Br, broccoli; S, squash; Co, cotton; P, pepper; E, eggplant; T, tomato.

### Isolation of *Salmonella* and STEC genes

The laboratory analysis was performed based on the methodology described by Topalcengiz et al. (2017). Briefly, to determine the prevalence of STEC, 25 mL of modified peptone water with pyruvate (mBPWp; Neogen, Lansing, MI, United States) was added to frozen filters and incubated at 35 ± 1°C for 24 h. For *Salmonella,* 100 μL of the pre-enrichment (mBPWp) was added into 10 mL of Rappaport-Vassiliadis (RV; Difco, Bectin, Dickinson, Sparks, MD, United States) and incubated at 42 ± 1°C for 48 h. Subsequently, DNA was extracted directly from mBPWp and RV using a DNeasy microbial kit (DNeasy^®^ UltraClean^®^ Microbial Kit, Qiagen, United States). Next, polymerase chain reaction (PCR) was performed to determine the presence of the markers for *Salmonella* (*invA*) and STEC (*hly, rfbE, flic, stx-*I*, stx-*II*, eaeA,* and *wzy*). The primer sequences used are listed in Table 2. *Salmonella* Braenderup (ATCC BAA-664) and *E. coli* O157:H7 (ATCC 43895) were used as positive and negative controls, respectively. A 50 mL PCR reaction for *Salmonella* and STEC markers consisted of 25 μL of Master Mix (GoTaq^®^Green Master Mix, Promega, Madison, WI, United States), 5 μL of each primer (reverse and forward), 5 μL of DNA template, and 10 μL of molecular biology grade water (Corning, Mediatech, Manassas, VA, United States). The PCR conditions for *invA* were: 1 min at 94°C for melting, followed by 35 cycles of 94°C for 1 min, 58.3°C for 30 s, and 72°C for 30 s with a final elongation of 72°C for 7 min, as previously described by Yanestria et al. (2019). For STEC, the PCR conditions were: 10 min at 95°C for melting, followed by 20 cycles of 94°C for 30 s, 59°C for 1 min with 0.5°C decreasing temperature per cycle, 72°C for 1 min, and a second 20 cycles of 94\u00B0C for 30 s, 49°C for 1 min, 72°C for 1 min and with a final elongation of 72°C for 7 min as previously described by Topalcengiz et al. (2017). For the gene *wzy,* the conditions used were the same as described by Iguchi et al. (2017): 25 cycles of 94°C for 30 s, 58°C for 30 s, and 72°C for 1 min. Lastly, gel electrophoresis was conducted on 1.5% agarose gel with 3 μL of ethidium bromide (Fisher Scientific, Fair Lawn, NJ, United States) 1X TAE buffer at 90 V for 35 min.

**Table 2:** Primer sequences used for PCR assay and expected sizes of the products.

Primers	5′➔3′ sequence	Size(bp)	Encoding region	References
invAF invAR	GTGAAATTATCGCCACGTTCGGGCAA TCATCGCACCGTCAAAGGAACC	285	Protein	Rahn et al. (1992), Topalcengiz et al. (2017)
hlyF hlyR	CCCTGGCAGACCTTTGATG CCGTGTCTTTTCTGATACTCA	773	Hemolysin	Manuel (2010)
rfbEF rfbER	GTGTCCATTTATACGGACATCCATG CCTATAACGTCATGCCAATATTGCC	292	O157 antigen	Hu et al. (1999)
flicF flicR	GCGCTGTCGAGTTCTATCGAGC CAACGGTGACTTATCGCCATTCC	625	Flagellar antigen H7	Hu et al. (1999)
stx-IF stx-IR	TGTAACTGGAAAGGTGGAGTATAC GCTATTCTGAGTCAACGAAAAATAAC	210	Shiga toxin 1	Hu et al. (1999)
stx-IIF stx-IIR	GTTTTTCTTCGGTATCCTATTCCG GATGCATCTCTGGTCATTGTATTAC	487	Shiga toxin 2	Hu et al. (1999)
eaeAF eaeAR	GACTGTCGATGCATCAGGCAAAG TTGGAGTATTAACATTAACCCCAGG	368	Intimin	Hu et al. (1999)
wzyF wzyR	CTC GAT AAA TTG CGC ATT CTA TTC CAA TAC GGA GAG AAA AGG ACC AA	106	O-antigen protein	Feng et al. (2021)

R, reverse; F, forward.

### *Salmonella* isolation and serotyping

*Salmonella-*positive samples on PCR were enriched following the FDA Bacteriological Analytical Manual (BAM) to obtain a culture for further serotyping (Andrews et al., 2023). Samples were enriched by streaked onto bismuth sulfite agar (BSA; Difco, Becton, Dickinson, Sparks, MD, United States), xylose-lysine-desoxycholate (XLD; Difco, Becton, Dickinson, Sparks, MD, USA), and hektoen enteric agar (HE; Difco, Becton, Dickinson, Sparks, MD, United States). XLD and HE were incubated at 35 ± 2°C for 24 h, and BSA was incubated at 35 ± 2°C for 48 h. Following incubation, if typical colonies were present, they were transferred onto triple sugar iron agar (TSI; Difco, Becton, Dickinson, Sparks, MD, United States) and lysine iron agar (LIA; Difco, Becton, Dickinson, Sparks, MD, United States) slants. For serotyping, isolates were streaked onto xylose-lysine-tergitol-4 agar (XLT4; Difco, Bectin, Dickinson, Sparks, MD, United States) and incubated at 35 ± 2°C for 24 h. All *Salmonella* isolates were also confirmed using a latex agglutination test (Oxoid, Hampshire, United Kingdom) and biochemical assays (urea, triple sugar iron agar, and lysine iron agar) before shipping isolates to the National Veterinary Services Laboratories (USDA, NVSL, Ames, Iowa, United States) for serotyping.

### Nutrient analysis for water

A 50 mL volume of each water sample was sent to the Soil, Forage, and Water Testing Laboratory at Auburn University for nutrient analysis. The analyzed nutrients included routine elements by ICAP (calcium, magnesium, potassium, phosphorous, copper, iron, manganese, zinc, boron, aluminum, cadmium, chromium, lead, sodium, nickel, and nitrate-n). The laboratory also provided information on water pH, soluble salts, and electrical conductivity. However, boron, cadmium, and chromium were below the limit of detection (<0.1) and were not included in the statistical analysis.

### Weather information collection

Weather information, including air temperature, solar radiation, and rain data, was obtained from the University of Georgia Weather Network.^1^ The closest weather stations to the ponds were located in Moultrie, Bowen, Tifton, and Ty Ty, GA.

### Statistical analysis

All data cleaning, visualization, and statistical analyses were performed using R Statistical Software [version 4.2.3 (2023-04-21 ucrt)]. All the analyses were performed for STEC virulence markers codetection, which indicated that virulence genes were present in the same sample (*stx-*I*, stx-*II, and *eaeA*). Logistic regression was implemented to determine the association of abiotic factors with the odds of codetection for virulent markers of STEC, and conditional forest analysis was further implemented to estimate whether those variables could be used to predict the codetection of STEC markers.

For logistic regression, all the numerical variables were scaled using the “caret” package as described by Belias et al. (2021). All the variables used for the analysis are listed in the Supplementary material (see Supplementary Tables 1, 2). To prevent multicollinearity in the logistic regression, a correlation matrix (see Supplementary Figure 1) using the “corrplot” package (Wei et al., 2021) was developed, and variables with strong correlation (>0.60) were omitted from the overarching model. In instances where two or more variables displayed significant correlation, one of them was systematically excluded. The global model for logistic regression included the following variables: rainfall before sampling (24 h, 48 h, and 7 days), generic *E. coli* (data not shown), phosphorus, nitrate, calcium, pH, humidity, and solar radiation. Additionally, pond and month of sampling were used as random effects to include temporal and special autocorrelation. To determine the best model, a stepwise regression was used based on AIC (Akaike Information Criterion). The best model was determined by the lowest AIC (72.5) and included the variables: calcium, nitrate, humidity, and solar radiation (see Table 3, Supplementary Table 3). The variance influence factor (VIF) was used to assess multicollinearity.

**Table 3:** Logistic regression analysis for the association between codetection of STEC virulence markers, nutrients in water, and environmental factors.

Outcome	Model variable	Coeff.	OR	95% CI	*p*-value
*Stx-*I*/stx-*II*/eaeA* codetection	Nitrate	1.32	3.75	1.45–23.12	0.09
	Calcium	−1.28	0.27	0.10–0.60	0.00 *
	Solar radiation	−0.90	0.40	0.14–0.98	0.06
	Humidity	−1.14	0.31	0.10–0.80	0.02 *

Coeff., beta coefficient from the logistic regression model; OR, odds ratio; CI, confidence interval.

**Figure 1:**
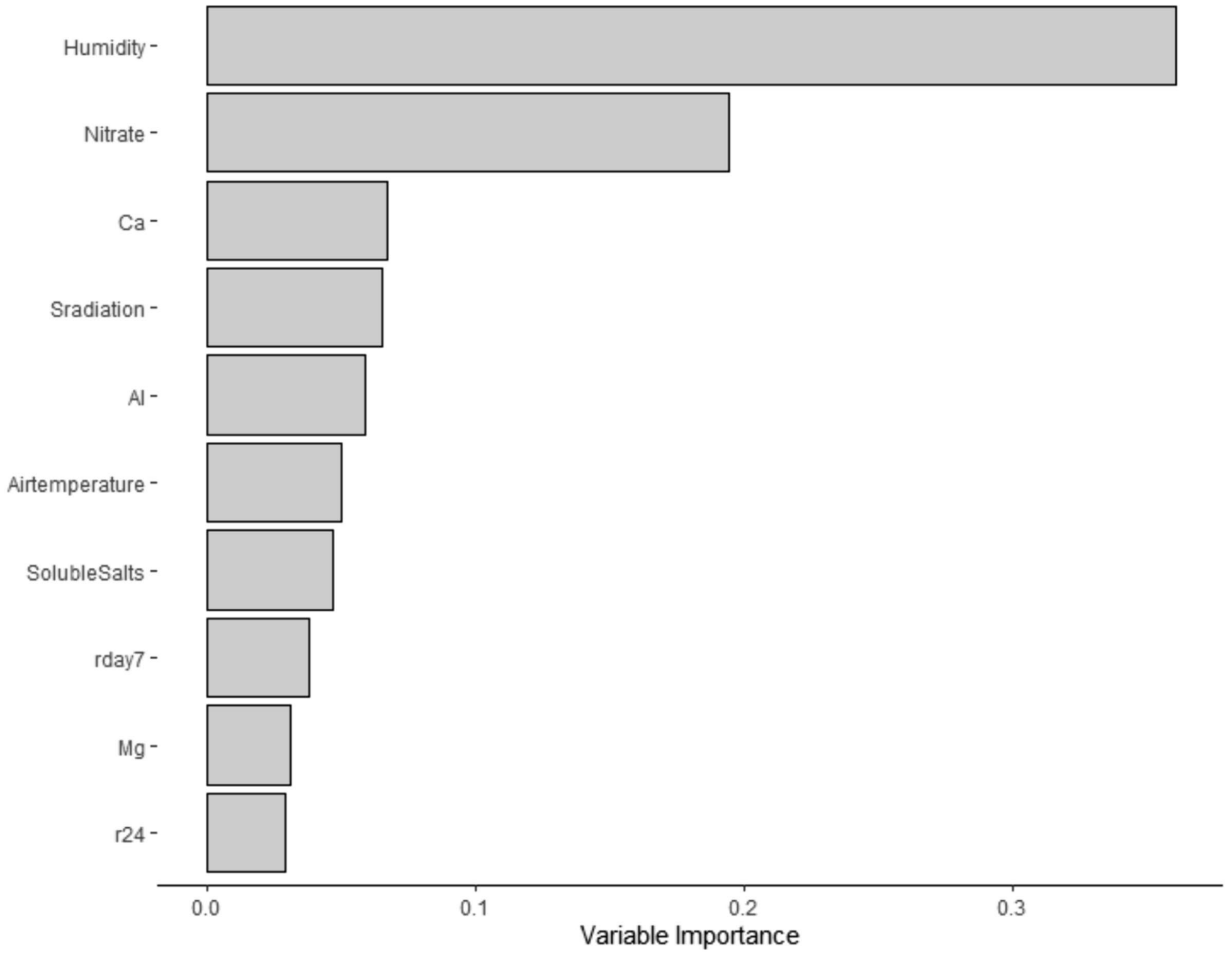
Top – ten predictors for STEC virulence markers for the conditional forest model.

Subsequently, using the “party” and “mlr” packages (Strobl et al., 2008; Belias et al., 2021; Hothorn et al., 2023), a conditional forest analysis was implemented. This analysis was conducted as an alternative option to predict STEC marker detection in water samples, as this analysis is robust to a large number of predictors and small sample sizes, as described by Belias et al. (2021). To maximize the area under the curve (AUC), repeated iterations of 10-fold cross-validation were used for tuning hyperparameters (mtry). For each forest, 20,000 trees were produced. Additionally, the variable of importance values was determined, and the top 10 predictors were extracted (Figure 1). Next, models were trained, tested, and cross-validated with our own data set. Although there are potential limitations of training the same data set, due to the nature of our experiment, this analysis represented a good fit, as we have various predictors and a small sample size.

## Results

Farms included in this study represent large vegetable-growing operations in the region. Crops grown on these farms included tomatoes, zucchini, kale, cabbage, watermelon, peppers, cotton, and eggplant (Table 1). A total of 88 samples were collected and analyzed as a part of the study. As a result, data obtained include serotypes of *Salmonella* (Table 4), the detection of STEC markers (Table 5), and the relationship with abiotic factors (Table 3).

**Table 4:** List of *Salmonella* serotypes isolated from surface water and list of tests applied to *invA* positive samples.

Sample information		From RV	Isolation and confirmation tests			NVSL^1^, USDA
Pond ID	Month	PCR	BAM^2^	XLT4	LATEX	Serotype
A3	May	−	+	+	+	Montevideo
A3	Jun	−	+	+	+	Saintpaul
B2	Jun	−	+	+	+	Mississippi
B4	Jun	+	+	+	+	Saintpaul
A4	Jul	−	+	+	+	Saintpaul
B1	Aug	−	+	+	+	Bareilly
B3	Sep	+	−	−	−	N/A*

1 NVSL, USDA: National Veterinary Service Laboratory.

2 Bacteriological Analytical Manual.

* N/A: No isolate was obtained.

**Table 5:** Percentages of STEC markers (%) for each Southwest Georgia pond over 11 months of sampling.

Pond ID	*stx-*I	*stx-*II	*eaeA*	*hly*	*rfbE*	*fliC*	Virulence markers*	All STEC
A1	9/11 (81.82%)	5/11 (45.45%)	5/11 (45.45%)	8/11 (72.73%)	5/11 (45.45%)	6/11 (54.55%)	3/11 (27.27%)	0/0 (0.00%)
A2	9/11 (81.82%)	5/11 (45.45%)	4/11 (36.36%)	10/11 (90.91%)	4/11 (36.36%)	6/11 (54.55%)	2/11 (18.18%)	0/0 (0.00%)
A3	10/11 (90.91%)	6/11 (54.55%)	2/11 (18.18%)	10/11 (90.91%)	4/11 (36.36%)	6/11 (54.55%)	2/11 (18.18%)	0/0 (0.00%)
A4	10/11 (90.91%)	6/11 (54.55%)	3/11 (27.27%)	9/11 (81.82%)	4/11 (36.36%)	6/11 (54.55%)	3/11 (27.27%)	0/0 (0.00%)
B1	10/11 (90.91%)	4/11 (36.36%)	2/11 (18.18%)	9/11 (81.82%)	3/11 (27.27%)	5/11 (45.45%)	2/11 (18.18%)	0/0 (0.00%)
B2	10/11 (90.91%)	5/11 (45.45%)	2/11 (18.18%)	10/11 (90.91%)	3/11 (27.27%)	9/11 (81.82%)	2/11 (18.18%)	0/0 (0.00%)
B3	8/11 (72.73%)	5/11 (45.45%)	4/11 (36.36%)	10/11 (90.91%)	4/11 (36.36%)	8/11 (72.73%)	2/11 (18.18%)	0/0 (0.00%)
B4	9/11 (81.82%)	5/11 (45.45%)	6/11 (54.55%)	11/11 (100%)	4/11 (36.36%)	8/11 (72.73%)	3/11 (27.27%)	0/0 (0.00%)
Total	75/88 (85.22%)	41/88 (46.59%)	28/88 (31.81%)	77/88 (87.50%)	31/88 (35.22%)	54/88 (61.63%)	19/88 (22%)	0/0 (0.00%)

* Virulence markers: *stx*-I, stx-II, and *eaeA*.

### Seasonal distribution of *Salmonella*

The overall prevalence of *Salmonella* in water samples was 6.81% (6/88 samples; Table 4). Four serovars were detected in 5 ponds (A3, A4, B1, B2, and B4). Pond A3 reported two serotypes of *Salmonella* (Montevideo and Saintpaul) for two consecutive months (May and June). *Salmonella* Bareilly and Mississippi were detected only once in ponds B1 and B2, respectively. A logistic regression was conducted; however, none of the parameters evaluated represented statistical significance (data not shown) for *Salmonella*. Discrepancies between methods were measured using PCR and culture-based methods. The PCR identified only two positive *Salmonella* samples, while culture-based methods identified 6 *Salmonella-*recovered samples.

### STEC markers distribution from irrigation ponds

All STEC markers were detected throughout the sampling period (February–December 2021), except for the gene *wzy* (Table 5). None of the samples had all seven genes present simultaneously; however, at least one of the six genes was detected in 97.72% of the samples. Each pond had the following individual STEC genes with the corresponding percentage of detection: *hly* (87.50%), *stx-*I (85.22%), *fliC* (61.63%)*, stx-*II (46.59%), *rfbE* (35.22), *eaeA* (31.81%), and *wzy* (0%). The frequency of individual genes varied among the samples, but the genes *stx*-I and *hly* were reported in over 70% of individual samples. The genes *eaeA* and *rfbE* were reported less frequently compared to the rest of the markers.

### Codetection of STEC virulence markers (*stx-*I, *stx-*II, and *eaeA*)

Twenty-two percent (19/88) of the samples were screened positive for the genes *eaeA* and *stx* (*stx-*I *and stx-*II) (Table 5). The occurrence of STEC virulence markers (*stx-*I*, stx-*II, and *eaeA*) was correlated with abiotic factors, including nutrients in water (i.e., calcium and nitrate), solar radiation, and humidity (Table 3). The likelihood of STEC virulence markers codetection in the ponds was negatively correlated with calcium and humidity. There was no other significant correlation between the STEC virulence markers and abiotic factors or fecal indicator bacteria (generic *E. coli*). The area under the curve for the logistic regression model was 0.86. In addition, a conditional forest analysis was used to predict the codetection of STEC virulence markers and the top ten ranked predictors based on the variable of importance values (Figure 1). Among the top ten predictors, the following categories of abiotic factors were included: nutrients (i.e., nitrate, calcium, aluminum, and magnesium), weather-related predictors (solar radiation, humidity, air temperature, rainfall events 24 h and 7 days prior to sampling), and one water physicochemical parameter (soluble salts). From those 10 predictors, our global model for logistic regression included at least seven variables; the remaining (soluble salts, air temperature, and magnesium) were not included to prevent multicollinearity. In addition, our “best model” determined by the stepwise regression was able to select the four most important variables obtained in the conditional forest analysis. The AUC for the conditional forest analysis was 0.9214.

## Discussion

This study was conducted to (i) investigate the prevalence of foodborne pathogens (i.e., STEC and *Salmonella*) in surface water sources from a large produce-growing area in the Southeastern United States and (ii) to determine the correlation among the prevalence of foodborne pathogens, fecal indicator bacteria (generic *E. coli*), and abiotic factors. Results provide insight into microbial water quality in Southwest Georgia, elucidating the use of abiotic factors as predictors of food safety hazards in surface agricultural water. A logistic regression was used to identify associations between abiotic factors and the presence of foodborne pathogens, and a conditional forest analysis was used as a prediction tool for the presence of STEC virulence markers. The results of this study can be further used to predict pathogen interaction with abiotic factors in subtropical environments.

Federal agencies like the Environmental Protection Agency (EPA) have established standards recommending the utilization of fecal indicator organisms like generic *E. coli* to determine microbial water quality. The Food Safety Modernization Act (FSMA) Produce Safety Rule previously stressed the use of microbial water testing by following EPA-approved methods, but with the new proposed Subpart E of the regulation, that is no longer a federal requirement, but it is still recommended as part of water assessment (US EPA, 2014; FDA, 2022). However, multiple studies have raised concerns about the poor correlation between generic *E. coli* and the presence of foodborne pathogens (Benjamin et al., 2013; Gu et al., 2013; Antaki et al., 2016; Weller et al., 2020b; Belias et al., 2021). Our results align with existing literature, as no correlation was measured between generic *E. coli* and the codetection of virulent genes for STEC and *Salmonella*, highlighting the limitations of solely relying on fecal indicators for pathogen incidence (Benjamin et al., 2013; Antaki et al., 2016; Falardeau et al., 2017; Topalcengiz et al., 2017; Liu et al., 2018; Gurtler and Gibson, 2022; Murphy et al., 2022; Xu et al., 2022). Findings in this study suggest that abiotic factors play a crucial role in the prevalence of foodborne pathogens and understanding them when conducting a holistic approach to agricultural water risk assessment is crucial.

### *Salmonella* findings: prevalence and serotypes

The identification of *Salmonella* in six out of the eight assessed ponds, either through culture or PCR, underscores the complexity and challenges encountered in environmental sample analysis. While the initial focus was on PCR for *Salmonella* detection, only two samples yielded positive results, prompting the adoption of the culture method. Despite the validation of the PCR reaction with a control (*Salmonella* Braenderup), negative results were observed, a common situation in environmental samples characterized by a diverse microbial community (Sipos et al., 2010). Plausible explanations for these findings include potential sample contamination during processing, primer cross-reaction, and the presence of low concentrations of targeted DNA. On the contrary, it is plausible to infer that samples exclusively identified through PCR may represent viable but non-culturable (VBNC) *Salmonella* or even dead cells, adding a layer of complexity to the interpretation of results. This can happen in water sources as the bacteria are stressed due to harsh conditions (Liu et al., 2018; Weller et al., 2020b; Stocker et al., 2022). For this study, no correlations were identified between the prevalence of *Salmonella* and the abiotic factors. However, all the *Salmonella* isolates were identified only in the summer months (May-Sept), where there is often an increase in air temperatures. Results are consistent with previous studies in the region regarding seasonality and temperature (Cooley et al., 2014; Luo et al., 2015; Murphy et al., 2022). The serotypes identified in the present study have previously been listed among the top 20 most reported serotypes associated with *Salmonella* infections in humans and have been previously isolated from water sources from Georgia (Luo et al., 2015; Antaki et al., 2016; Harris et al., 2018; Deaven et al., 2021) and other states (Truitt et al., 2018). The prevalence of *Salmonella* in the ponds was substantially lower when compared to previous studies in Georgia and surrounding states; for instance, we isolated *Salmonella* from 8% (7/88) of the samples by filtering a volume of 150 mL of water, as previously described. In contrast, studies in southern Georgia reported higher rates, but these were observed in larger water volumes, with 11.0% (34/285) of samples containing *Salmonella*, each sample having a total of 880 mL of water (Antaki et al., 2016), and 49% (52/107) in studies where a total volume of 1.8 L was analyzed (Harris et al., 2018). However, the type of surface water might impact the microflora. For example, rivers, lakes, or ponds have different water flows and geological characteristics. In the Mid-Atlantic region of the United States, researchers reported a higher prevalence of *Salmonella* in water collected from rivers compared to ponds (Sharma et al., 2020), which may justify the lower numbers of *Salmonella* isolates detected in the ponds evaluated in the current study.

Additional factors such as land use, the presence of wild animals, and concentrated animal feeding operations (CAFO) are relevant factors for microbial contamination (Strawn et al., 2013b). As surface water is exposed to the environment and animals are more susceptible to microbial contamination, wild animal activity (e.g., birds, reptiles, wild hogs) was observed in all the ponds’ surrounding areas, which may have contributed to the ponds’ microflora (Rocha et al., 2022). Poultry operations are often with a source of *Salmonella* and *Campylobacter jejuni* (Vereen et al., 2013; Bardsley et al., 2021). Considering that poultry is one of the major commodities in Georgia (Kane, 2023), it is possible that the prevalence of *Salmonella* in Georgia could be attributed to the presence of such operations. In our study, at least three poultry farms were within 5–8 km of pond A and at least 5 km from pond B1.

### Detection of Shiga toxin-producing *Escherichia coli*

*E. coli* O157:H7 might have been present in the water sources as 6/88 samples had *rbfE* and *stx* (*stx*I and *stx*II) genes simultaneously. Detecting both *stx* and O157 markers is a good indicator of the potential presence of *E. coli* O157:H7 (Franz et al., 2007; Feng et al., 2021). Studies conducted in southern Georgia isolated *E. coli* O157:H7 in surface water (Jenkins et al., 2012; Gu et al., 2013). STEC is frequently associated with ruminants, mainly with cattle (Jay et al., 2007; Jenkins et al., 2011; Munns et al., 2015). None of the farms in this study had animal operations within the farm, and the closest cattle operation was 7 km from Pond A and at least 5 km from Pond B1. STEC can also be associated with human fecal markers (Weller et al., 2020b). All evaluated ponds had either houses or packinghouses within less than 500 m of proximity, which may have impacted the water quality. The other genes were detected throughout all the sampling time and in different percentages (Table 4), but *hly* (77/88, 87.5%) and *stx*-I (75/88, 85.22%) were found with more frequency, indicating the presence of Shiga toxin (*stx-*I) and hemolysin which can also be found in other pathotypes of *E. coli*, including extraintestinal pathogenic *E. coli* (Wyborn et al., 2004). When comparing all the genes for STEC, the genes reported with less frequency were *eaeA* (28/88), followed by *rbfE* (31/88). This differs from the finding of Haymaker et al. (2019), who measured STEC genes in 10 liters of water and reported that the gene *eaeA* (88/510) was the most frequently detected in water sources from the Mid-Atlantic region of the United States.

Although this study exclusively aimed to detect specific STEC virulence markers without utilizing culture-based methods or whole-genome sequencing (WGS) to isolate and identify individual bacterial strains due to their complexity, this approach may limit the interpretation of PCR signals, especially in cases involving multiple serotypes. Therefore, it is crucial to exercise caution when interpreting PCR results, particularly in scenarios with the potential presence of multiple serotypes. For future studies, researchers can improve the accuracy and reliability of findings by integrating complementary techniques such as bacterial isolation and WGS, thereby facilitating a more comprehensive understanding of microbial populations and their associated virulence factors.

### Codetection of STEC virulence markers (*stx-*I, *stx-*II, and *eaeA*)

The codetection of *eaeA* and *stx* genes (I and II) was evaluated in this study to determine the presence of Shiga toxin-producing *E. coli* genes. Although *E. coli* O157:H7 is frequently reported with contaminating produce, it was not confirmed in this study. Other non-O157 serogroups (i.e., O111, O26, O117, O121, and O145) can also produce Shiga toxins, carry virulence genes, and have been previously detected in surface water (Nadya et al., 2016). A recent study from the CDC found that consuming raw produce like lettuce and tomatoes is the leading cause of non-O157 outbreaks (Marder et al., 2023). In the past, *E. coli* O26:H11 has caused outbreaks and hemolytic-uremic syndrome (HUS; Alharbi et al., 2022). The virulence genes for STEC (*eaeA* and *stx*) have been primarily associated with patients with severe life-threatening complications with HUS (Eklund et al., 2002; Werber et al., 2008). STEC, when compared to other foodborne pathogens, has a lower infectious dose that results in high morbidity and mortality (Saxena et al., 2015). In the present study, 19/88 (21.59%) of collected samples were PCR-screened positive for the virulence genes. Similar results were reported by Belias et al. (2021), who reported a 21% (36/169) prevalence from canal water. Research conducted in agricultural water from New York, Arizona, and California identified different percentages of codetection, respectively, 2.7% (16/588), 48% (44/83), 88% (77/88), 57% (188/330) (Strawn et al., 2013a; Cooley et al., 2014; Weller et al., 2020b). It is relevant to emphasize that the use of the simultaneous detection of those genes without a culture or serotyping in a single sample may lead to an overestimation of STEC prevalence, as it has been suggested that their presence might indicate the presence of either a single organism possessing both genes or multiple organisms, each with one of the genes (Weller et al., 2020b; Belias et al., 2021). Thus, careful consideration of the limitations associated with gene detection methods is crucial in accurately assessing the prevalence of STEC, highlighting the need for complementary techniques such as culture or serotyping in future studies to distinguish between single organisms with both genes and multiple organisms.

Based on the logistic regression model, the odds of codetection of virulence genes of STEC were negatively associated with calcium and humidity (*p*-value >0.05). The best model failed to identify any substantial correlations with either nitrate or solar radiation (p-value >0.05). Findings in our study suggest that for each ppm increase in calcium, the odds of codetection decreased significantly (*p* < 0.05). The odds ratio (OR) of 0.27 indicates that higher calcium levels reduce the odds of codetection by 73%. The average calcium levels were 13.08 ppm with a median of 12.70 and a maximum of 41 ppm. Literature often associates the levels of calcium with oxidative stress in bacteria, the increase in calcium also can impact the lipid bilayer of *E. coli* (Dominguez, 2004) and biofilm formation (Bilecen and Yildiz, 2009; Hu et al., 2022). No documented literature was found on the codetection of virulent factors and their relationship with calcium concentrations in water used for irrigation.

There was a negative correlation between relative humidity and STEC virulence factors. The median relative humidity during the study was 79.08%, with a minimum of 63.25% and a maximum of 89.40%. Higher humidity levels were associated with a significant decrease in the odds of *stx-aeaA* codetection by a factor of 0.31. Limited information is available on the effect of relative humidity and pathogens in water sources. In a study conducted in Spain, authors did not find correlations between relative humidity and levels of *E. coli* in water sources (Weller et al., 2020a; Truchado et al., 2021), on the other hand, suggested that the average of relative humidity was classified as an important factor for *Listeria monocytogenes* isolation from water sources. In non-aquatic environments opposite findings have been reported suggesting that higher environmental moisture could enhance pathogen survival in soil, especially in subtropical and tropical environments (Zhang et al., 2010; Liu et al., 2013). While it is widely recognized that higher moisture levels generally promote pathogen survival, particularly in terrestrial environments, our findings suggest a more nuanced relationship within aquatic ecosystems. In aquatic environments, factors such as water flow, temperature fluctuations, and microbial interactions can significantly influence the survival and proliferation of pathogens. Therefore, while our results may seem contradictory to the general understanding of humidity’s effects on pathogen survival, they may reflect the complex interplay of various environmental factors unique to surface water ecosystems. This highlights the importance of considering the specific conditions of aquatic environments when interpreting microbial dynamics and virulence marker distribution.

Although solar radiation and nitrate were not statistically significant (p-value >0.05), biological significance can be inferred as our findings suggest that solar radiation was negatively associated with the codetection of virulent genes. The mean and median for solar radiation from this data set were 17.32 MJ/m^2^ and 16.97 MJ/m^2^, respectively. With each unit (MJ/m2) increase in solar radiation, the odds of codetection were reduced by approximately 60%. Previous studies have reported that solar radiation inactivates *E. coli* in water (Whitman et al., 2004; Kim et al., 2023). A study in Northeast Georgia reported similar results for generic *E. coli* but established that STEC (O157:H7) strains were resistant to solar radiation (Jenkins et al., 2011). Studies in New York and New Zealand report negative correlations between *E. coli* and solar radiation (Weller et al., 2020a; Bunyaga et al., 2023). Weller et al. (2020a, b) reported that in Arizona, the likelihood of codetection of *eaeA*-*stx* increased as solar radiation increased. Previous findings suggest that UV rays can impact *E. coli* as a function of water turbidity given by suspended particles, as higher concentrations of suspended particles reduce the UV rays’ penetration and impact on pathogens (Tousi et al., 2021). Further research must be conducted to determine biological interactions between solar radiation and foodborne pathogens in aquatic environments. Nitrate, on the other hand, was considered the second most important variable in our conditional forest analysis, and similarly to other nutrients like phosphorous, the literature suggests that this nutrient has biological significance in the presence of foodborne pathogens in water sources (Gu et al., 2013). Although not considered to be statistically significant according to our logistic regression analysis, it might represent biological significance as for each unit increase in nitrate, the odds of codetection increased by a factor of 3.37. In our study, the mean concentration for nitrates was 2.2 ppm, with a maximum of 19.1 ppm. Phosphorus and nitrogen are crucial nutrients for eutrophication processes, (US EPA, 2013). A cyanobacteria-like organism was occasionally observed in some of the ponds, which may have supported the growth of STEC and *Salmonella,* as they can form biofilms on aquatic plants or attach to suspended solids in aquatic ecosystems (Chekabab et al., 2013; Cho et al., 2022). Additional abiotic factors like physicochemical parameters are well known to impact waterborne bacteria, including water pH (Rodrigues et al., 2020), turbidity, water temperature, and conductivity have been previously associated with the presence of pathogens like *L. monocytogenes* and *Salmonella* (Chung et al., 2020); however, the outcomes from this study did not find a significant correlation between STEC virulence factors and other abiotic factors.

## Conclusion

This study provides insight into the role of abiotic indicators in detecting microbial contamination in agricultural water sources located in the Southeast United States. Furthermore, significant associations between calcium and humidity are key factors for the codetection of virulence markers for STEC. This study reinforces the poor correlation between generic *E. coli* and foodborne pathogens. Further studies must be conducted to determine the serogroups of STEC present in surface water used for irrigation. Our study also suggests that although all the markers for *E. coli* O157:H7 were not detected simultaneously in a single sample, markers were detected throughout the study which might indicate the potential presence of other dangerous *E. coli* (enteropathogenic, enterohemorrhagic, STEC). The presence of *Salmonella* and STEC in agricultural water poses a public health concern as several outbreaks have been associated with the serovars detected at the evaluated farms.

## Data availability statement

The raw data supporting the conclusions of this article will be made available by the authors, without undue reservation.

## Author contributions

ZC: Data curation, Formal analysis, Methodology, Validation, Writing – original draft. LD: Conceptualization, Methodology, Visualization, Writing – review & editing. AS: Conceptualization, Data curation, Visualization, Writing – review & editing. CR: Conceptualization, Funding acquisition, Investigation, Methodology, Project administration, Resources, Supervision, Visualization, Writing – review & editing.

## Footnotes

^1^ http://georgiaweather.net/
