# Supplementary material for: Prevalence of STEC virulence markers and Salmonella as a function of abiotic factors in agricultural water in the southeastern United States
Source: Front Microbiol. 2024 May 20;15:1320168. doi: 10.3389/fmicb.2024.1320168 (PMC11144861; doi:10.3389/fmicb.2024.1320168)
Supplement: Supplementary file 1 [file Data_Sheet_1.docx]

**SUPPLEMENTAL INFORMATION**

**Table S1.** Description of factors used in regression and random forest analyses.

| Factor Type | Factor | Units |
| --- | --- | --- |
| Microbial water quality | *E. coli* level | Log_10_ MPN/ 150 mL |
| Physicochemical parameters | pH  Electrical conductivity  Soluble Salts | NA  mmhos/cm  ppm |
| Nutrients | Magnesium (Mg)  Phosphorous (P)  Aluminum (Al)  Barium (Ba)  Cupper (Cu)  Iron (Fe)  Manganese (Mn)  Sodium (Na)  Niquel  Lead (Pb)  Calcium (Ca)  K  Zink (Zn)  Nitrate | ppm |
| Weather | Avg Air temperature day of sample collection | °C |
|  | Avg solar radiation day of sample collection | mJ/m^2^ |
|  | Precipitation 48 h before sample collection | mm |
|  | Precipitation 24 h before sample collection | mm |
|  | Precipitation 7 days before sample collection | mm |
|  | Relative Humidity Day of sample collection | % |

^a^All weather data was collected from the University of Georgia Weather Network (<http://georgiaweather.net/>). The closest weather station to each point was used to create weather factors.

**Table S2.** Summary statistics of factors used in regression and random forest analyses.

| Predictor | Min^a^ | Q1 | Median | Mean | Q3 | Max^a^ |
| --- | --- | --- | --- | --- | --- | --- |
| Avg air temperature day of sample collection | 11.28 | 16.46 | 21.71 | 20.79 | 25.82 | 27.34 |
| Avg solar radiation day of sample collection | 8.76 | 12.23 | 16.97 | 17.32 | 21.56 | 27.01 |
| Precipitation 48 h before sample collection | 0.00 | 0.00 | 0.25 | 17.44 | 17.44 | 161.50 |
| Precipitation 24 h before sample collection | 0.00 | 0.00 | 0.00 | 4.04 | 4.04 | 48.51 |
| Precipitation 7 days before sample collection | 0.00 | 0.00 | 0.00 | 1.30 | 1.30 | 15.75 |
| Avg Relative Humidity Day of sample collection | 63.25 | 74.06 | 79.08 | 78.22 | 83.44 | 89.40 |
| pH | 6.41 | 7.35 | 7.54 | 7.49 | 7.66 | 8.20 |
| Electrical conductivity | 0.09 | 0.18 | 0.22 | 0.22 | 0.25 | 0.48 |
| Soluble Salt | 56 | 119 | 143 | 142 | 159 | 310 |
| Mg | 1.4 | 4.15 | 5.45 | 5.49 | 6.92 | 11.5 |
| P | 0.1 | 0.1 | 0.1 | 0.14 | 0.1 | 1.1 |
| Al | 0.1 | 0.1 | 0.1 | 0.25 | 0.1 | 2.5 |
| Ba | 0.1 | 0.1 | 0.1 | 0.12 | 0.1 | 1.1 |
| Cu | 0.1 | 0.1 | 0.1 | 0.13 | 0.1 | 1.1 |
| Fe | 0.1 | 0.1 | 0.1 | 0.18 | 0.1 | 1.6 |
| Mn | 0.1 | 0.1 | 0.1 | 0.14 | 0.1 | 1.5 |
| Na | 4 | 7.75 | 9.5 | 9.713 | 11.125 | 21.9 |
| Ni | 0 | 0.1 | 0.1 | 0.098 | 0.1 | 0.1 |
| Pb | 0 | 0.1 | 0.1 | 0.14 | 0.1 | 1.5 |
| Ca | 3.6 | 9.22 | 12.7 | 13.08 | 16.35 | 41 |
| K | 2.9 | 6.97 | 9.1 | 10.68 | 11.22 | 47.6 |
| Zn | 0 | 0.1 | 0.1 | 0.14 | 0.1 | 1.5 |
| Nitrate | 0.7 | 1.1 | 1.55 | 2.2 | 2.22 | 19.1 |

^a^Min= minimum, Max= maximum

**Table S3.** Summary statistics for continuous water quality factors used in regression analysis.

| Factor | Min^a^ | Q1 | Median | Mean | Q3 | Max^a^ |
| --- | --- | --- | --- | --- | --- | --- |
| Calcium | 3.60 | 9.22 | 12.70 | 13.08 | 16.35 | 41 |
| Nitrate | 0.70 | 1.10 | 1.55 | 2.20 | 2.22 | 19.10 |
| Humidity | 63.25 | 74.06 | 79.08 | 78.22 | 83.44 | 89.40 |
| Solar radiation | 8.76 | 12.23 | 16.97 | 17.32 | 21.56 | 27.01 |

^a^Min=minimum, Max=maximum

**
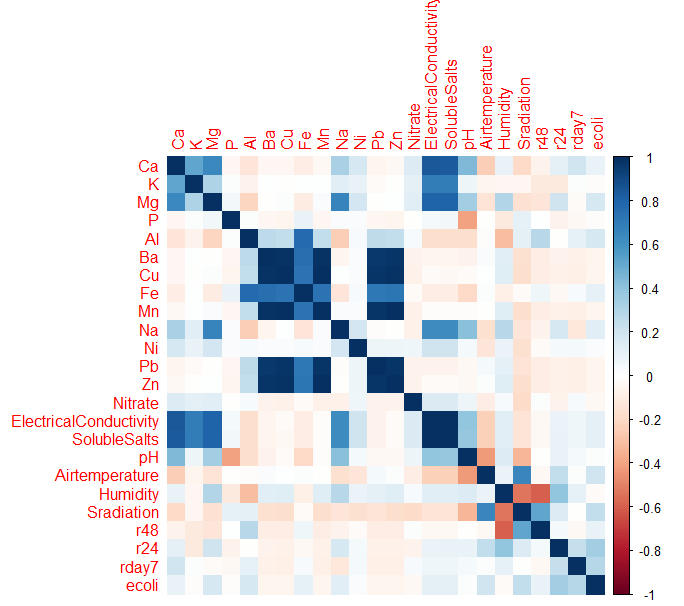
**

**Figure S1.** Correlation Matrix
